# Long-Term Adherence to Benralizumab and Sustained Clinical Benefits in Patients with Severe Eosinophilic Asthma: Insights from GALERNA, a Retrospective Real-World Study in Spain

**DOI:** 10.3390/jcm15124564

**Published:** 2026-06-12

**Authors:** Ismael García-Moguel, Juan Agüero Calvo, Diana Betancor Pérez, José Ángel Carretero Gracia, Rocío Magdalena Díaz-Campos, Carmen Fernández Martínez de Septien, Ana Gómez-Bastero Fernández, Yurena Hernández Galván, Juan Antonio Lloret-Queraltó, Inmaculada Lluch Tortajada, Nuria Marina Malanda, Cristina Martín-García, Álvaro Martínez Mesa, Eva Martínez-Moragon, Juan Francisco Medina Gallardo, Alicia Padilla-Galo, Gerardo Pérez Chica, Inmaculada Plasencia García, Patricia Prieto Montaño, Carolina Puchaes Manchón, David Romero Ribate, Marcela Valverde-Monge, María Soledad Zamarro Parra, Jose Luis Sanchez-Trincado, Javier Nuevo, Elisa Luzon, José Luis Velasco-Garrido

**Affiliations:** 1 Allergology Department, Hospital 12 de Octubre, 28041 Madrid, Spain; 2 Pulmonology Department, Hospital Universitario Marqués de Valdecilla, 39008 Santander, Spain; 3 Allergology Department, Hospital Fundación Jimenez Diaz, 28040 Madrid, Spain; 4 Pulmonology Department, Hospital Royo Villanova, 50015 Zaragoza, Spain; 5 Pulmonology Department, Hospital 12 de Octubre, 28041 Madrid, Spain; 6 Pulmonology Department, Hospital Universitario de Burgos, 09006 Burgos, Spain; 7 Pulmonology Department, Hospital Virgen Macarena, 41009 Seville, Spain; 8 Pulmonology Department, Hospital Insular Gran Canaria, 35016 Las Palmas de Gran Canaria, Spain; 9 Pulmonology Department, Hospital Viladecans, 08840 Barcelona, Spain; 10 Pulmonology Department, Hospital de la Ribera, 46600 Valencia, Spain; 11 Pulmonology Department, Hospital Universitario de Cruces, 48903 Barakaldo, Spain; 12 Allergology Department, Hospital Clínico Universitario Salamanca, 37007 Salamanca, Spain; 13 Pulmonology Department, Hospital Universitario Virgen de la Victoria, 29010 Málaga, Spain; 14 Pulmonology Department, Hospital Universitario Dr. Peset, 46017 Valencia, Spain; 15 Pulmonology Department, Centro de Especialidades Doctor Fleming, 41005 Seville, Spain; 16 Pulmonology Department, Hospital Universitario Médico Quirúrgico Ciudad de Jaén, 23007 Jaén, Spain; 17 Pharmacy Department, Hospital Nuestra Señora de la Candelaria, 38010 Santa Cruz de Tenerife, Spain; 18 Pulmonology Department, Complejo Hospitalario Universitario de Albacete, 02006 Albacete, Spain; 19 Pulmonology Department, Hospital Universitario Juan Ramón Jiménez, 21005 Huelva, Spain; 20 Pulmonology Department, Hospital Universitario La Paz, 28046 Madrid, Spain; 21 Allergology Department, Hospital Reina Sofía de Murcia, 30003 Murcia, Spain; 22 Medical Department, AstraZeneca Farmacéutica, 28050 Madrid, Spain

**Keywords:** severe eosinophilic asthma, benralizumab, severe asthma exacerbation, real-world evidence

## Abstract

**Background**: The GALERNA study is a retrospective real-world observational study conducted across 21 hospitals in Spain, aiming to evaluate long-term clinical benefits and adherence to benralizumab in 255 adult patients with severe eosinophilic asthma (SEA) over a follow-up period of up to 144 weeks. **Objectives**: Primary objectives focused on assessing adherence to benralizumab, while secondary objectives included the description of severe asthma exacerbation rates, systemic corticosteroid (SCS) use, persistence to benralizumab, lung function, asthma control, and the proportion of patients achieving super-response or clinical remission. Data were collected at baseline (48 weeks prior to benralizumab initiation/index date), follow-up 1 (FUP1) (0–48 weeks), FUP2 (49–96 weeks), and FUP3 (97–144 weeks) after the index date. **Results**: At baseline, patients demonstrated a substantial disease burden characterised by impaired lung function, poorly controlled asthma, and frequent severe exacerbations. The results indicated high adherence rates to benralizumab, with 92.9% of patients receiving each of the prescribed doses of benralizumab at week 48 and 70.6% at week 144. Patients showed substantial and sustained clinical improvements, with a reduction in the proportion of individuals presenting at least one severe exacerbation from baseline to FUP3 and a 74% decrease in SCS use for the same period. Lung function also improved, with the proportion of patients achieving pre-bronchodilator FEV_1_ ≥ 80% rising to 52% at 144 weeks. Furthermore, mean Asthma Control Test (ACT) scores increased to 20.5, with 68.5% of patients achieving well-controlled asthma (ACT ≥ 20). By the end of the study, 63.6% of patients achieved super-response and 39.1% showed clinical remission, with an overall benralizumab persistence of 79.8% during all follow-up periods. **Conclusions**: The GALERNA study provides compelling real-world evidence that benralizumab affords marked and sustained clinical benefits together with high long-term adherence in Spanish SEA patients, reinforcing its usefulness as a long-term therapeutic option in routine clinical practice.

## 1. Introduction

Asthma is a chronic, heterogeneous respiratory disease characterised by inflammation of the airway, causing its swelling and narrowing, and giving rise to symptoms such as dyspnoea, wheezing, cough, chest tightness, and shortness of breath [1,2,3].

Severe eosinophilic asthma (SEA) is the most prevalent phenotype among severe asthma (SA) cases [4,5], driven primarily by type 2 (T2) inflammation in which eosinophils play a central role. SEA is typically associated with airway and blood eosinophilia, increased peripheral IgE levels, high fractional exhaled nitric oxide (FeNO), and frequent comorbidity, particularly in the form of chronic rhinosinusitis with nasal polyps (CRSwNP) [4,6,7].

The addition of biologics in SEA treatment came as a valuable option, targeting key mediators of eosinophilic inflammation [8], helping to address the clinical challenges associated with this asthma phenotype, and preventing the associated adverse events of corticosteroids. Benralizumab is one of the currently available biologics approved for the treatment of SEA; it is a humanised IgG1k monoclonal antibody of murine origin that binds to the alpha subunit of the interleukin-5 receptor (IL-5Rα) expressed by cells such as eosinophils and basophils [9,10]. Benralizumab promotes rapid and near-complete depletion of eosinophils from both the peripheral blood and airway [11,12,13]. Indeed, the efficacy and safety of benralizumab in SEA were demonstrated in three pivotal phase 3 trials: SIROCCO (ClinicalTrials.gov NCT01928771), CALIMA (ClinicalTrials.gov NCT01914757), and ZONDA (ClinicalTrials.gov NCT02075255) [14,15,16]. These studies showed that, compared to placebo, benralizumab significantly reduced the exacerbation rates, improved lung function and asthma symptoms, and decreased or even eliminated daily OCS use. These benefits have been shown to persist for up to five years with long-term use [17].

Although real-world data on the long-term outcomes of SEA patients treated with benralizumab in Spain are limited, the ORBE I and ORBE II studies laid critical groundwork for benralizumab in the real-life setting. ORBE I showed that after initiating treatment with benralizumab, patients experienced improved asthma control, quality of life, and reduced exacerbation rates, including in individuals refractory to other anti-IL-5 biologics [18]. In turn, ORBE II, the largest Spanish cohort before the current GALERNA study, confirmed these sustained benefits over one year, reporting reductions in exacerbations and OCS use, together with improvements in lung function [19,20].

Despite the clinical benefits demonstrated by biologics such as benralizumab, poor adherence remains a significant barrier to optimal disease control. Adherence rates to biological therapies are generally higher than to traditional treatments, often exceeding 70%, since such therapies are administered less frequently and have fewer systemic side effects than corticosteroids [21,22]. However, various factors can still influence adherence to biologics, including patient perceptions, fear of injections, socioeconomic barriers, and healthcare system limitations [23,24]. Improving adherence to biologics in the context of SEA is increasingly recognised as crucial for maximising therapeutic outcomes, reducing exacerbation rates, and minimising healthcare costs [25], particularly in real-world clinical settings.

Thus, there is a specific need for studies examining the evolution of patients receiving biologic therapies in both home and clinical settings, with a focus on adherence. Such research is crucial for understanding how consistent biologics use impacts exacerbation rates, disease control, and quality of life. In addition, very few studies to date have monitored the evolution of patients beyond one year of treatment with biologics in a real-world setting.

GALERNA is an observational, retrospective cohort study designed to describe long-term adherence in SEA patients treated with benralizumab in routine clinical practice in Spain. Despite the growing use of biologic therapies for SEA, real-world evidence evaluating long-term outcomes beyond the first year of treatment remains limited, particularly regarding treatment adherence and persistence. While several real-world studies have demonstrated the short-term effectiveness of benralizumab, data describing sustained adherence, long-term persistence, and the maintenance of clinical benefits over extended follow-up periods are scarce. The GALERNA study was therefore designed to address this gap by providing real-world data on adherence, persistence, and long-term clinical outcomes in a large Spanish cohort of patients with SEA treated with benralizumab, with follow-up extending to nearly three years. By integrating adherence metrics with multiple clinical outcomes over prolonged follow-up, GALERNA adds novel insight into the durability of benralizumab treatment in routine clinical practice. Collecting these real-world data will enhance our understanding of how SEA patients on benralizumab treatment evolve and will contribute to future strategies for disease monitoring and control.

## 2. Methods

### 2.1. Study Design and Patient Population

An observational, retrospective cohort study was carried out to describe adherence during the long-term follow-up of SEA patients treated with benralizumab according to clinical practice in Spain. The study was conducted using data from the Departments of Pulmonology, Allergy, and Hospital Pharmacy of 21 hospitals in Spain.

The participating investigators enrolled patients aged 18 years or older who had been diagnosed with SEA by a physician and started benralizumab treatment between 1 October 2019 and 31 March 2022, as per routine clinical practice in Spain. Eligible patients had received at least one dose of benralizumab and had available medical records covering the 48 weeks preceding the index date (defined as the date of the first benralizumab injection) and at least 48 weeks following the index date. Patients concurrently participating in any other interventional clinical trial involving asthma treatments during either the baseline period or the follow-up period were excluded.

The baseline period covered the 48 weeks prior to the index date. The follow-up period (FUP) was defined as up to 144 weeks after the index date and was divided into FUP1 (0–48 weeks), FUP2 (49–96 weeks), and FUP3 (97–144 weeks) after the index date.

### 2.2. Study Treatment

The decision to prescribe benralizumab was under the discretion of the physician and was made prior to patient inclusion in the study. Thus, an observational period in a past time frame was chosen to ensure that benralizumab was prescribed according to clinical practice.

### 2.3. Compliance with Ethical Guidelines

The study was carried out in accordance with the Declaration of Helsinki and Good Clinical Practice Guidelines, and applicable regulatory requirements. The study protocol was approved by the Independent Ethics Committee (IEC) of Hospital Universitario 12 de Octubre (Madrid, Spain) (CEIm reference 23/051). Informed consent was not required for this retrospective observational study because it made use of anonymised health data already recorded in electronic health records, posed minimal risk to participants, and the obtaining of consent was not feasible. This exemption is justified by the specifications of the Council for International Organisations of Medical Sciences and the World Health Organisation; Regulation (EU) 2016/679 of the European Parliament and of the Council, of 27 April 2016, on the protection of natural persons with regard to the processing of personal data and the free circulation of these data; Organic Law 3/2018, of 5 December, on the Protection of Personal Data and guarantee of digital rights; and Spanish Royal Decree 957/2020, of 3 November, provided the study was approved by a Research Ethics Committee. Data from all participating sites were combined into a single anonymised dataset for analysis. AstraZeneca maintained confidentiality standards through the assignment of a unique subject identification number for each patient enrolled in this study.

### 2.4. Study Outcomes

The primary objective was to assess adherence to benralizumab over a period of up to 144 weeks in SEA patients treated with benralizumab according to clinical practice in Spain. Adherence was assessed through the proportion of prescribed injections the patient actually received during their individual exposure time to benralizumab.

Secondary objectives were to describe the baseline characteristics of patients with SEA who start treatment with benralizumab, to evaluate patient follow-up and persistence to benralizumab throughout the study, and to estimate the severe asthma exacerbation rate. Patient follow-up was defined as the length of treatment, in days, from the first administration to either the end of the follow-up period or until treatment discontinuation. Severe asthma exacerbations were defined according to the 2009 American Thoracic Society (ATS)/European Respiratory Society (ERS) consensus [26] and measured as annual rates both at baseline and during follow-up. Additionally, the study aimed to determine the proportion of patients achieving super-response and clinical remission during the observation period. Super-responders were defined by no exacerbations and no use of any systemic corticosteroids (SCS), while clinical remission corresponded to zero exacerbations, no use of any SCS (cycles or maintenance), Asthma Control Test (ACT) score ≥ 20, and pre-bronchodilator (pre-BD) forced expiratory volume in one second (FEV_1_) improvement ≥ 100 mL from the index date [27,28]. For the assessment of super-response and clinical remission, only patients with available data for all individual components required for each composite outcome were considered evaluable. Consequently, the number of patients included in the analyses of super-response and clinical remission may differ, depending on the availability of simultaneous data for exacerbations, systemic corticosteroid use, asthma control, and lung function variables. SCS exposure was described as the median daily dose, expressed in prednisone equivalents. When reported, cumulative annual SCS doses were calculated by multiplying the median daily dose by 365 days.

Exploratory objectives involved describing healthcare resources utilisation (HCRU), patient-reported outcomes (PROs) (ACT, and Test of Adhesion to Inhalers (TAI)) [29,30,31] and evaluating the use of SCS in the cortico-dependent patients subgroup. A cortico-dependent patient was defined as a subject who had received continuous SCS for at least 6 months in the last year or had been on continuous SCS for at least three months before the index date. Temporary and permanent benralizumab discontinuation dates during the study follow-up were also recorded, including the main reasons leading to discontinuation.

### 2.5. Data Collection

Study data were collected from existing medical records and hospital pharmacy registers according to routine clinical practice. Individual patient data were collected and pseudo-anonymised in an electronic database designed specifically for this study.

Data collected retrospectively at baseline comprised demographic information, smoking habits, asthma-related comorbidities, SCS use, previous biological therapies, PROs (ACT and TAI), efficacy variables including lung function (forced vital capacity (FVC), FEV_1_, and FEV_1_/FVC), and inflammatory biomarkers (eosinophil count and total IgE in peripheral blood and fractional exhaled nitric oxide (FeNO)). The data collected during FUP included information on benralizumab administration, temporary and permanent benralizumab discontinuations, SCS use, PROs, and other efficacy variables.

### 2.6. Statistical Analysis

The sample size was determined to evaluate the primary objective, ensuring that the secondary objectives were also met. The proportion of patients with severe asthma who adhere to benralizumab treatment over long-term follow-up in clinical practice is not known; the principle of maximum variability (p = q = 50%) was thus used to calculate the sample size. The study estimated a required sample size of approximately 300 patients, achieving a maximum error of ±5.8%, and ensuring robust precision for the obtained estimators.

Quantitative variables were described using measures of central tendency and dispersion (mean, standard deviation (SD), median, minimum, maximum, first quartile (Q1), and third quartile (Q3)), and the results were expressed as the mean (SD) or median (interquartile range Q1–Q3). Qualitative variables, in turn, were reported as absolute and relative frequencies.

For the main study outcome, benralizumab adherence rate was measured as the proportion of prescribed injections actually received (based on prescribed dosage information extracted from pharmacy dispensing data) during the study period. Complementarily, it was tracked whether benralizumab administration was in a hospital setting or self-administered.

Patient follow-up to benralizumab treatment was defined as the length of treatment (in days) during the study period, from first administration to the end of the follow-up period, or to treatment discontinuation during the follow-up period (whichever occurred first). The severe asthma exacerbation rate and the proportion of patients who achieve both benralizumab super-response and clinical remission were also analysed. PROs (ACT and TAI) were presented descriptively as a change in scores from baseline (the closest measure prior to the index date) to the closest measurement (when available) for each follow-up period. Lastly, persistence to benralizumab was measured as the proportion of patients without permanent benralizumab discontinuations during the study and its follow-up periods.

## 3. Results

### 3.1. Baseline Characteristics of the Study Population

A total of 255 evaluable patients were analysed. Demographic and clinical data were collected at baseline. Patients had a mean (SD) age of 56.4 (13.1) years, 67.7% were women, and the mean (SD) body mass index (BMI) was 28.7 (5.9) kg/m^2^. The mean (SD) age at asthma diagnosis was 38.7 (17.5) years, and 50.6 (14.5) at severe asthma diagnosis, with 47.3% of the patients presenting allergic asthma according to the criterion of the physician (Table 1). The majority were non-smokers (62.1%), and former smokers constituted 34.2%. Nearly all patients (99.6%) had comorbidities, the most common being CRSwNP and rhinitis (37.6% and 34.1%, respectively), followed by gastroesophageal reflux and arterial hypertension (each 26.3%). A total of 10.9% of the patients used SCS to manage comorbid conditions.

The median [Q1–Q3] eosinophil count was 405 [200–600] cells/µL, while the median [Q1–Q3] FeNO value was 35 [19–65] parts per billion (ppb) (Table 2). A total of 100 patients (39.4%) had previously received one or more biological therapies: 72% used omalizumab, 44% mepolizumab, and 2% reslizumab. The main reasons for discontinuing previous biological treatments were lack (47.2%) or loss (34.7%) of response. Use of SCS (as cycles or maintenance) was recorded in 71.3% of patients, with a median [Q1–Q3] daily dose of 1.6 [0.7–3.8] mg of prednisone equivalents, which corresponds to a cumulative annual dose of 584.4 mg. A total of 14.9% of patients were cortico-dependent, with a median daily dose of 6.9 [3.5–17.6] mg, and a cumulative annual dose of 2.52 g of prednisone equivalents. Additionally, 70.4% of patients used combinations of inhaled corticosteroids with long-acting beta-agonists and long-acting muscarinic antagonists (ICS + LABA + LAMA), while 66.1% used leukotriene receptor antagonists (LTRA).

In the year prior to starting benralizumab, more than half of the patients (58.8%) experienced severe exacerbations, and 56.9% used SCS for treating them (Table 2). Following exacerbations, 30.2% registered emergency room (ER) visits, 10.2% hospital admissions, and one patient (0.4%) required intensive care unit (ICU) admission. Only 33.6% of the patients presented FEV_1_ ≥ 80%, indicating substantially impaired lung function in approximately two-thirds of the patient population. The mean (SD) ACT score was 13.8 (5.6) at baseline, with only 19.1% of patients having well-controlled asthma (ACT ≥ 20) (Table 2).

### 3.2. Patient Follow-Up

The number of patients with available data during each follow-up period was 255 during the first 48 weeks after benralizumab initiation (FUP1), 237 during weeks 49–96 (FUP2), and 211 during weeks 97–144 (FUP3). The patients had a median [Q1–Q3] follow-up time of 336.0 (322.0–350.0) days during FUP1, 673.0 (651.0–696.0) days during FUP2, and 1001.5 (922.5–1024.5) days during FUP3.

### 3.3. Adherence to Benralizumab

**Figure 1:**
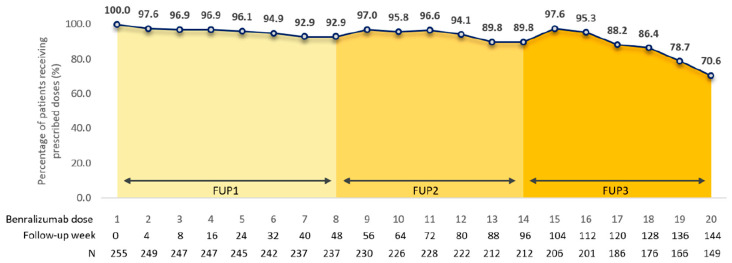
Percentage of patients (%) who, according to pharmacy dispensing data, received the prescribed weekly dose in accordance with the approved scheduled use. FUP: follow-up period.

Figure 1 shows the percentage of patients who received the timely scheduled doses according to benralizumab approved use (every 4 weeks for the first 3 doses, and every 8 weeks thereafter [32]), which corresponded to 92.9% (n/N = 237/255), 89.8% (n/N = 212/236) and 70.6% (n/N = 149/211) at weeks 48, 96 and 144 after benralizumab initiation, respectively (Figure 1). Overall, the mean (SD) patient adherence rate to benralizumab during the 144-week follow-up period was 96.0% (11.1) (n = 211), while the proportion of patients with ≥80% adherence during each follow-up period (FUP1-FUP3) was 91.0% (n = 232), 94.9% (n = 223), and 98.6% (n = 208), respectively. Most patients (99.6%) received their first two doses (Weeks 0 and 4) in a controlled hospital setting (Figure S1). Benralizumab administration transitioned then to home settings, reaching 50.4% by Week 16. This trend remained consistent at 77–81% from Weeks 104 to 144, with minimal fluctuations through the end of the 3-year follow-up (Figure S1).

### 3.4. Severe Asthma Exacerbations and Related Healthcare Resources Utilisation

**Figure 2:**
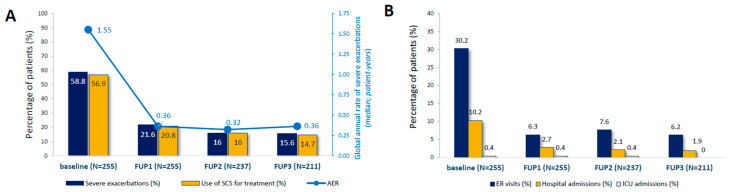
Severe asthma exacerbations and related HCRU throughout the study period. (**A**) Percentage of patients (%) with severe asthma exacerbations, with use of systemic corticosteroids (SCS) for their treatment and global annual rates of severe exacerbations (AER) (median; patient-years). (**B**) Percentage of patients (%) who reported ER visits, hospital, and ICU admissions due to severe asthma exacerbations. FUP: follow-up period.

At baseline, more than half of the patients presented severe exacerbations (58.8%), with a global annualised rate of 1.55 exacerbations per patient-year, and 56.9% of these exacerbations were accompanied by treatment with SCS. These values were reduced to 21.6% (patients with severe exacerbations) and 20.8% (treated with SCS) after 48 weeks of benralizumab, and to 15.6% and 14.7%, respectively, after being treated for 144 weeks. In turn, up to 35.7% of the patients (n = 91) remained exacerbation-free during the whole 144-week follow-up of the study. The global annual rate of severe exacerbations registered after 48 weeks of benralizumab was 0.36 and remained constant over the subsequent follow-up periods (Figure 2A). At baseline, a total of 38 patients required hospitalisations due to exacerbations (n/N = 38/249; 15.3%). Hospital admissions due to severe exacerbations decreased from 10.2% to 1.9% of the patient population between baseline and 144 weeks, respectively. In terms of ER visits due to exacerbations, 30.2% of the patients required such visits at baseline, in contrast to 6.2% after 144 weeks of benralizumab. No ICU admissions due to exacerbations occurred at FUP3, with only one patient (0.4%) requiring such admission in the FUP1 and FUP2 periods (Figure 2B).

Concerning annual rates, the patients presented 1.85 ER visits and 1.05 hospital admissions per patient-year. These rates decreased to 0.45 (ER visits) and 0.44 (hospital admissions) at FUP1. At FUP3, hospital admissions and ER visits reached a rate of 0.06 and 0.19, respectively (Figure S2A). The median duration of hospitalisations at baseline was 3.0 days (n = 33), and decreased to 0.0 days during FUP1, FUP2, and FUP3 (n = 31, n = 29, and n = 25, respectively). Patients registered for asthma specialist care had a global annual rate of 4.45, while for general practitioner care, the rate was 3.57. Visits to both care categories declined by FUP1 (asthma specialists 3.19 and general practitioners 1.22). The global annual rate for asthma specialists was 2.91 and 3.03 at FUP2 and FUP3, respectively, while the rate for general practitioners was 1.32 and 1.07, respectively, for the same follow-up periods (Figure S2B).

### 3.5. Lung Function and T2 Biomarkers

**Figure 3:**
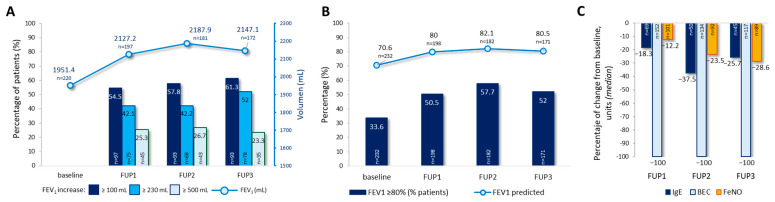
Lung function and T2 biomarkers throughout the study period (**A**) pre-BD FEV1 (mL) and percentage of patients with an increase in ≥100, 230, and 500 mL in FEV1 (**B**) FEV1 predicted (%) and percentage of patients (%) with FEV1 ≥ 80% (pre-BD). (**C**) Percentage of reduction in median IgE levels (IU/mL), blood eosinophil count (BEC, cells/µL), and FeNO (ppb) from baseline. FUP = follow-up period.

In relation to lung function, the mean pre-BD FEV_1_ increased from baseline during the 48 weeks after benralizumab initiation and thereafter. More than half of the patients registered an increase in ≥100 mL from baseline in all follow-up periods (54.5%, 57.8%, and 61.3%, respectively) and, more importantly, 25.3% presented a substantial increase in FEV_1_ from baseline of ≥500 mL during FUP1 and 23.3% during FUP3 (Figure 3A). Regarding predicted values, the mean pre-BD FEV_1_ (%) increased from 70.6% to 80.0%, 82.1%, and 80.5% during FUP1, FUP2, and FUP3, respectively. A total of 33.6% of patients had FEV_1_ ≥ 80% predicted at baseline, in contrast to 50.5%, 57.7%, and 52% during FUP1-FUP3 (Figure 3B).

Regarding T2 biomarkers, eosinophil counts showed a marked and sustained reduction throughout the study, declining from a median of 405 cells/µL to undetectable levels at all follow-up points, corresponding to a −100% reduction from baseline levels. In comparison to baseline, there were also reductions in −18.3%, −37.5%, and −25.7% in the median total IgE levels (IU/mL), and progressive decreases in −12.2%, −23.5%, and −28.6% in the median FeNO values during FUP1, FUP2, and FUP3, respectively (Figure 3C).

### 3.6. Systemic Corticosteroid Use

**Figure 4:**
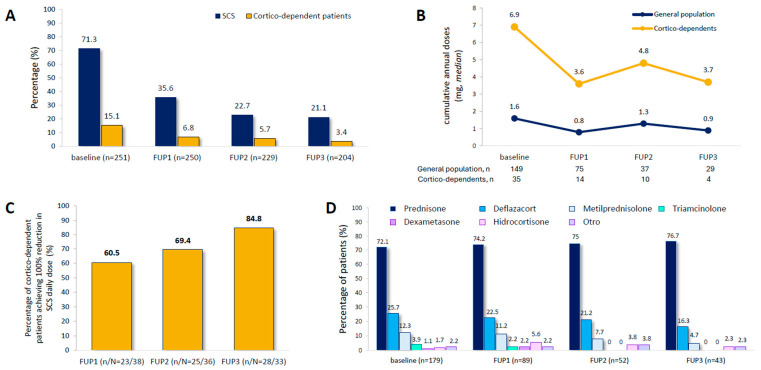
Use of systemic corticosteroids (SCS). (**A**) Percentage of patients (%) using SCS and cortico-dependent patients. (**B**) Cumulative median annual doses (mg) of SCS in the general and cortico-dependent population (**C**). Proportion of cortico-dependent patients achieving a 100% reduction in SCS daily dose from baseline (**D**). Percentage of patients (%) using different types of SCS during the study follow-up periods. FUP = follow-up period; SCS = systemic corticosteroids.

The percentage of patients reporting any SCS use (cycles and/or maintenance) was 71.3% at baseline and decreased to 35.6% and 21.1% after 48 and 144 weeks, respectively (Figure 4A). The median daily dose of any SCS use in the general population was reduced from 1.6 mg (cumulative annual 584.4 mg) at baseline to 0.8 mg (cumulative annual 292.2 mg) after 48 weeks and remained similar (0.9 mg) during FUP3 (Figure 4B).

The proportion of cortico-dependent patients decreased from 15.1% (n = 38) one year before benralizumab to 3.4% (n = 7) after 144 weeks of treatment (Figure 4A). Moreover, for this group, the median daily SCS doses were reduced from 6.9 mg (cumulative annual 2.52 g) at baseline (n = 38) to 3.7 mg (cumulative annual 1.35 g) in those patients that remained cortico-dependent at FUP3 (n = 7) (Figure 4B). The proportion of cortico-dependent patients who achieved a 100% reduction in SCS daily dose from baseline was 60.5% (n = 23) versus 84.8% (n = 28) after 48 and 144 weeks of treatment, respectively (Figure 4C). Only two patients (n/N = 2/248; 0.8%) and one patient (n/N = 1/227; 0.4%) needed to start SCS treatment after 48 and 96 weeks of benralizumab, respectively, and none required such medication at the last follow-up.

Regarding specific corticosteroid agents, the use of prednisone remained comparable before and throughout benralizumab therapy, while the percentage of patients treated with deflazacort and methylprednisolone decreased from 25.7% and 12.3% at baseline to 16.3% and 4.7% after 144 weeks of treatment, respectively (Figure 4D).

### 3.7. Patient Reported Outcomes

**Figure 5:**
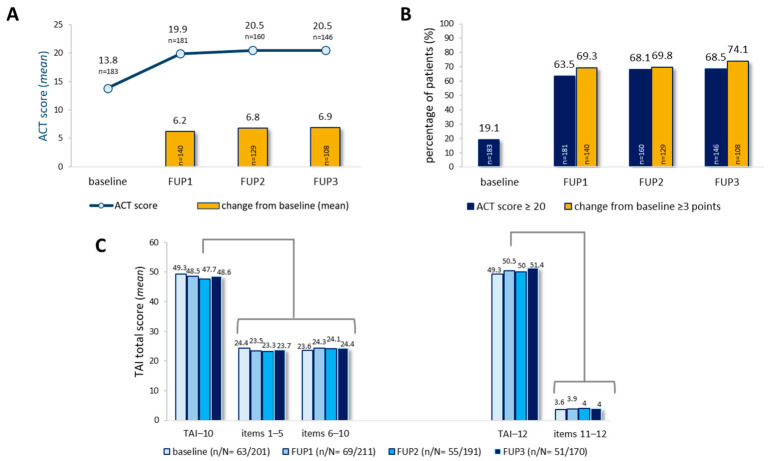
(**A**) Total score for Asthma Control Test (ACT) and the mean change from baseline score are shown. (**B**) Percentage of patients (%) with well-controlled asthma (ACT ≥ 20) and presenting a minimal clinically important difference (MCID) ≥ 3 points from baseline assessments (**C**). The mean total score for the Test of Adhesion to Inhalers (TAI) is shown. FUP: follow-up period.

At baseline, the mean (SD) ACT score was 13.8 (5.6), with an increase to 19.9 (5.1) after 48 weeks and to 20.5 (4.9) after both 96 and 144 weeks following the start of benralizumab (Figure 5A). The mean change from baseline was 6.2 points at FUP1, 6.8 points at FUP2, and 6.9 points at the last visit. Initially, only 19.1% of the patients had an ACT score ≥ 20 at baseline, although the percentage increased to 63.5%, 68.1%, and 68.5% at FUP1, FUP2, and FUP3, respectively. Notably, 63.5% of the patients showed a clinically relevant increase in ACT score of ≥3 after 48 weeks of treatment, and this figure increased to 74.1% after 144 weeks (Figure 5B).

Regarding adherence to inhaled therapy measured by the TAI questionnaire, the total scores remained relatively similar across baseline and the three FUP (Figure 5C). Notably, scores for both the full TAI-10 and TAI-12 were consistently high throughout the study period.

### 3.8. Super-Response and Clinical Remission

**Figure 6:**
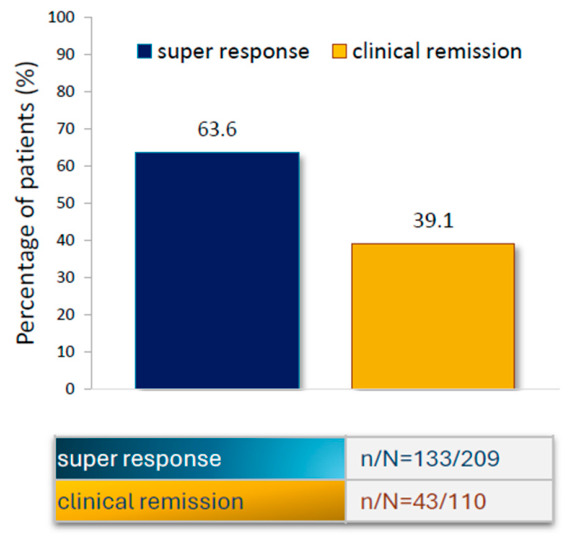
Super response and clinical remission after long-term follow-up (FUP3, up to 144 weeks). Super response was defined as the absence of exacerbations and of any use of SCS, whereas clinical remission required, apart from both previous criteria, an ACT score ≥ 20 and a lung function improvement of ≥100 mL in pre-BD FEV1. Super-response and clinical remission were analysed only in patients with available data for all the above-mentioned criteria.

A total of 61.4% (n = 154) and 63.5% (n = 148) of the patient population exhibited a super-response (no exacerbations and no SCS use) during FUP1 and FUP2, respectively, while clinical remission (super-response plus ACT ≥ 20 and pre-BD FEV_1_ increase ≥ 100 mL) was achieved by 22.5% (n = 31) and 36.1% (n = 43) during FUP1 and FUP2, respectively. At long-term (during FUP3), super-response was achieved by 63.6% (n = 133) and clinical remission by 39.1% (n = 43) of the evaluated patients (Figure 6).

### 3.9. Benralizumab Discontinuation

**Figure 7:**
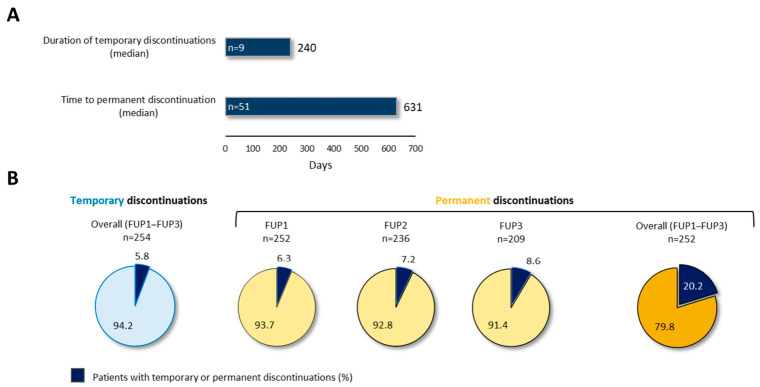
Benralizumab discontinuations throughout the study period. (**A**) Median duration of temporary discontinuations and time registered from benralizumab initiation to permanent discontinuation for the overall population (in days). (**B**) Percentage of patients (%) with temporary and permanent discontinuations registered at different follow-up visits, and overall benralizumab persistency. FUP: follow-up period.

Temporary discontinuations of benralizumab (i.e., patients who resumed the treatment in the course of study follow-up) were reported in 5.8% of the patients (n = 14) during the whole follow-up period (Figure 7A). The mean duration of temporary discontinuations for the overall population was a median [Q1–Q3] of 240.0 [152.0–459.0] days, representing approximately 34.3 weeks (Figure 7B). The proportion of patients reporting permanent benralizumab discontinuation was 6.3% (16/255), 7.2% (17/236), and 8.6% (18/209) during FUP1, FUP2, and FUP3, respectively, implying overall benralizumab persistence during all the follow-up periods of 79.8% (n/N = 201/252) (Figure 7A). The median [Q1–Q3] time registered from benralizumab initiation to permanent discontinuation was 631.0 [364.0–823.0] days (Figure 7B). The main reasons for temporary and permanent discontinuations are described in Table S1.

## 4. Discussion

The GALERNA study evaluated 255 patients with SEA treated with benralizumab as per routine clinical practice during a maximum follow-up of 144 weeks after the start of benralizumab, assessing adherence to treatment and the evolution of asthma clinical outcomes. This study is among the first to systematically evaluate adherence to biologics in severe asthma, with a focus on benralizumab. Furthermore, it represents the largest Spanish cohort with a long-term follow-up to date, addressing a significant gap in real-world data on the long-term outcomes of patients with SEA.

At baseline, patients exhibited substantial disease burden; almost all of them had comorbidities (e.g., CRSwNP, rhinitis), a late onset of asthma, biomarker levels indicating eosinophilic inflammation, and ACT scores suggesting poorly controlled asthma. Lung function was severely impaired (FEV_1_ ≥ 80% in only 33.6% of the cases), and 58.8% experienced severe exacerbation pre-treatment, with one third of the patients reporting ER visits. Benralizumab treatment resulted in substantial clinical improvements, and these benefits were sustained up to week 144. In relation to asthma control, the mean ACT scores increased by 6.2 and 6.9 points during FUP1 and FUP3, respectively, with respect to baseline. Similarly, lung function improved, with the proportion of patients achieving FEV_1_ ≥ 80% being 50.5% and 52% at the same timepoints. These clinical gains were accompanied by a favourable decrease in inflammation biomarkers, most notably a depletion of eosinophil count, along with a slight reduction in total IgE and FeNO levels. These benefits translated into high proportions of patients meeting super-response (63.6%) and clinical remission (39.1%) criteria after three years of treatment. Such findings suggest that for Spanish SEA patients in a routine clinical setting, benralizumab treatment provides clinical benefits that are evident during the first 48 weeks and are sustained throughout the almost three-year follow-up period.

The assessment of real-world adherence to biologics is crucial to understand patient needs, since poor adherence often leads to suboptimal asthma control. In this study, the proportion of patients receiving each of the prescribed doses of benralizumab was high (92.9% at week 48) but declined moderately for the last recorded doses (70.6% at week 144). This is consistent with real-world studies of biologics, where the adherence rates are usually lower than in clinical trials [33,34]. To our knowledge, there are no other studies on benralizumab adherence with a three-year follow-up period. Our results closely match those from the ANANKE study (90% adherence at 96 weeks), with 89.8% of patients receiving the prescribed scheduled dose according to the pharmacy dispensing data [35], and are comparable to those reported for other biologics at 52 weeks (with retention ranging from 60% to 75%) [36]. Indeed, based on the data of this study, adherence to benralizumab may apparently outperform that of other biologics such as dupilumab (65% adherence at 2 years) [36,37] and omalizumab (70.5% at 1 year) [38], particularly when prescribed in a two-week regimen (65% at 5 years) [39]. The greater convenience of benralizumab dosing (first three doses every 4 weeks and thereafter every 8 weeks) may be an added factor to its efficacy and could positively prompt patients to continue scheduled doses in comparison to other biologics [40].

Asthma exacerbations are important contributors to the burden of SEA, also requiring treatment with SCS, and involving hospitalisation and/or ER visits, and accelerating lung function decline [41]. A 74% reduction in exacerbations (58.8% to 15.6% of patients) was reported after benralizumab administration for 144 weeks, as well as a decrease in the proportion of patients using SCS to treat them, with the global annual rate of severe exacerbations kept at a low and stable level of 0.3–0.4 exacerbations per patient-year. In the three-year Italian study published by Pini et al., benralizumab achieved an 89% reduction in exacerbation rate at 36 months, closely paralleling the decrease observed in the GALERNA study over a similar period, and consistent with persistent annual rates as low as 0.26–0.43 per year [42].

The improvement in mean ACT scores (+6.9 points from baseline to 144 weeks) reflects better control of SEA symptoms and is aligned with the results of the ORBE II study, which reported a +7.4 points increase at one year [19]. The proportion of patients achieving ACT ≥ 20 increased to 63.5% at 48 weeks and to 68.5% at 144 weeks—this being similar to the 73.8% reported by the ORBE II study at one year, suggesting sustained clinical benefit [20]. In further support of these findings, long-term real-world studies of SEA patients with benralizumab and mepolizumab have shown improvements in asthma control. In a 4-year study of benralizumab [43], the mean (SD) ACT score increased from 14.57 (2.45) at baseline to 24.52 (0.75) at 48 months, with over 70% of patients achieving asthma control (ACT ≥ 20) at 48 months. In relation to mepolizumab, the REDES study reported an increase in mean (SD) ACT score from 14.63 (5.16) at baseline to 21.09 (3.72) at 12 months [44]. Regarding the minimal clinically important difference (MCID) of ≥3 points in mean ACT score, 72.1% of patients in the ORBE II study reached this threshold after one year [20], and the GALERNA study yielded a similar outcome, with 74% of the patient population reaching this goal after 144 weeks. These data emphasise the sustained, clinically relevant benefits of biological therapies on asthma control in SEA.

Lung function also improved, as the percentage of patients with pre-BD FEV_1_ ≥ 80% increased from 33.6% at baseline to 52% after 144 weeks. These results are comparable to those reported by Martinez-Moragon et al. in a cohort of patients treated with benralizumab during 12 months (51%) [45]. Padilla-Gallo et al. indicated that achieving FEV_1_ ≥ 80% may be challenging in some patients and could indicate more compromised lung function [20]. Several factors may contribute to irreversible airway damage and structural changes [46,47], especially the duration of asthma, thus supporting the idea that early intervention is essential for preserving lung function [48,49]. Nonetheless, marked gains were observed in the lung function volumes in most of the GALERNA study patients. Indeed, up to 25.3% of them presented an FEV_1_ increase of ≥500 mL at 48 weeks and 23.3% at 144 weeks after the start of benralizumab.

Eosinophil depletion to a median of 0 cells/µL throughout the entire follow-up period is consistent with the antibody-dependent cytotoxicity mechanism of benralizumab, and aligns with findings from the BORA extension trial [50]. Blood IgE levels showed a slight decrease after 144 weeks. Contoli et al. reported a 36% reduction in total IgE levels already after a shorter period (108 ± 4 days) of treatment with benralizumab, but with no effect in the case of mepolizumab [51]. IgE is a key regulator mediating T2 inflammatory response by interacting with IgE receptors in effector cells [52], and its decline may alleviate SEA symptoms. Patients also exhibited a median reduction in −11 ppb (−28.6%) in FeNO. The ORBE II study showed a median reduction from 36.8 to 24.9 ppb over a median follow-up of 19.5 months with benralizumab [20]. Although it has been shown that baseline FeNO does not predict the response to benralizumab [53], individuals with baseline FeNO ≥ 75 ppb presented a substantial decrease from 100 ppb (88–145) to 58 ppb (33–102) at one year of treatment with benralizumab—a finding not consistently registered across studies with mepolizumab [53,54]. This effect may be attributable to the mechanism of action of benralizumab, targeting IL-5R, while mepolizumab and reslizumab target IL-5 [55]. Consequently, benralizumab not only depletes eosinophils but also acts on other cells that express IL−5Rα, which may indirectly impact other immune pathways beyond that of IL-5 [56,57].

The proportion of patients using SCS substantially decreased from baseline to 144 weeks of treatment, as did the cumulative annual doses of SCS for asthma treatment. Moreover, the median cumulative annual dose of SCS for treating severe exacerbations was halved in the general population and was reduced 100% in over 80% of the cortico-dependent patients after 144 weeks of benralizumab. These findings align with those of the ORBE II study, where following benralizumab, 82.5% of the patients in the overall population were free of maintenance OCS use, and the mean daily dose of OCS in the cortico-dependent population showed a reduction of 70.4% [20]. Regarding mepolizumab, in patients requiring maintenance OCS in the REDES study, the mean daily dose decreased by 59.9% after 12 months, and up to 47.8% of these patients totally eliminated OCS use—this being similar to the 43% and 57% reported in the REALITI-A study after 1 and 2 years of treatment, respectively [54,58]. Taken together, these data highlight the sustained efficacy of benralizumab in securing long-term corticosteroid withdrawal, suggesting the importance of prolonged eosinophil depletion as a valuable strategy to spare SCS use in severe eosinophilic asthma.

The 63% super-response rate at 144 weeks exceeds the rates reported by real-world studies from Kavanagh and Jackson et al. [27,59] (39% and 57.2%, respectively). This rate is more comparable to that of the XALOC-1 programme [60] (60%) and the multicentre RE-ASGRAMUR Group study (63%) [61], both of which also assessed benralizumab after 48 weeks. Achievement of a sustained super-response (60% at 48 weeks and 63% at 144 weeks) suggests a potential for sustained disease modification. Regarding clinical remission, 22.5% of the patients reached this status at 48 weeks, which is lower than the percentage reported by Padilla-Gallo et al. (ORBE II study) over the same time period [20]. In addition, the longer duration of our study might capture relapses not observed in shorter studies. The increase in remission rate in the longer term cannot be attributed mostly to the later persistence of patients who respond well, as not many presented with benralizumab discontinuation. Nonetheless, patient follow-up for up to almost three years is of utmost importance, in line with the new REMAS (Spanish Consensus on Remission in Asthma) concept of assessing remission after three years of treatment [7]. Our clinical remission rate at 144 weeks (39.1%, n/N = 43/110) matches the rates recently reported by Pelaia (42.1%; n/N = 69/164) [62] after two years of treatment and by Martinez-Moragon et al. after one year (44.1%; n/N = 41/93) [45]. In relation to the latter, it should be noted that the criteria for clinical remission in both studies were the same, with the exception of the parameter FEV_1_, which their study defined as a value of ≥80% [45]. Admittedly, these findings should be interpreted with caution, given the observational study design and the lack of universally standardised definitions for clinical remission and super-response. Nevertheless, the fact that our results consistently align with other real-world benralizumab cohorts suggests that these rates are a robust reflection of the treatment benefits in clinical practice.

Additionally, the low benralizumab discontinuation rates observed in this study may also reflect the substantial and long-lasting clinical improvements observed in the patients, as well as be indicative of the safety profile of benralizumab. Temporary discontinuations were reported in only 5.8% of the patients overall, while permanent discontinuations occurred in 20.2% of all patients in the study over the entire follow-up period. This rate is consistent with that recorded in the BREEZE study, with a benralizumab discontinuation rate of only 1.3% after approximately one year [63], or with the 16% and 10.9% discontinuation rates also observed with benralizumab after two years in the XALOC-1 study [60] and after 96 weeks in the ANANKE study [35]. Furthermore, the observed rate is also lower than the 27% discontinuation rate reported for mepolizumab after a shorter period of two years in the REALITI-A study [54] or the 41% discontinuation rate observed for dupilumab in a retrospective study after a three-year follow-up period [64]. Despite the absence of data regarding adverse events in this study, these low discontinuation rates are coherent with the known safety profile of benralizumab, which has been extensively evaluated in dedicated extension trials, including the MELTEMI study, in which benralizumab was well tolerated for up to five years, with no increase in serious adverse events, serious infections, or malignancies compared with earlier treatment periods [65].

Some other limitations should be considered when interpreting the study data. In its observational nature, this study was designed to collect information available in routine clinical practice; hence, the validity of the results was limited by the information available and the presence of missing data. Additionally, a single-arm study limits causal inferences without comparison against a control group or a standard of care treatment. The analysis was purely descriptive, lacking inferential statistical testing; consequently, the results should be interpreted as exploratory. Concerning adherence, we relied on pharmacy dispensing records rather than administration dates, since the latter were available only for a limited number of patients. The study is also subject to potential selection and attrition bias, as the requirement for a minimum of 48 weeks of follow-up and the reduction in sample size over time may have overestimated long-term effectiveness and adherence. Finally, formal subgroup analyses stratifying patients by previous biologic use or by specific clinical phenotypes (such as allergic status) were not performed in the present work. To the best of our knowledge, the long-term (three-year) follow-up of this study provides robust real-world evidence on the impact of benralizumab upon several asthma outcomes, from eosinophilic inflammation biomarkers to lung function and the dependence on corticosteroids for managing symptoms despite their known associated adverse effects. The study is also strengthened by the involvement of a multidisciplinary team that included pulmonologists, allergists, and professionals from both nursing and pharmacy, thus facilitating a well-rounded and integrated approach to the research.

## 5. Conclusions

In summary, the GALERNA study provides compelling real-world evidence that clinical benefits were observed and sustained in patients with severe eosinophilic asthma who received benralizumab during long-term treatment. Over 144 weeks, patients experienced marked improvements in asthma control and lung function, with reductions in exacerbations, corticosteroid use, and inflammatory biomarkers. Furthermore, the adherence rates were comparable to or exceeded those reported for other biologics. Notably, a large proportion of patients achieved super-response and clinical remission, suggesting that benralizumab may support sustained disease control in a substantial proportion of patients in routine clinical practice. These data reinforce the value of benralizumab as a long-term therapeutic option for the management of SEA and highlight the importance of ongoing real-world studies to assist in clinical decision-making and optimise patient outcomes.

## Figures and Tables

**Table 1:** Baseline sociodemographic characteristics of the study population (48 weeks before benralizumab).

Parameters	Total (N = 255)
**Age**, years ^a^	56.4 (13.1)
**Women**, n/N (%) ^a^	172/254 (67.7)
**BMI** (kg/m^2^) ^b^	28.7 (5.9)
**Age at asthma diagnosis**, years ^c^	38.7 (17.5)
**Age at SEA diagnosis**, years ^d^	50.6 (14.5)
**Allergic asthma**, n/N (%) *^,e^	116/245 (47.3)
**Smoking history**, n/N (%) ^f^	
Non-smoker	151/243 (62.1)
Former smoker	83/243 (34.2)
Smoker	9/243 (3.7)
**Comorbidities**, n/N (%)	254/255 (99.6)
CRSwNP	96 (37.6)
Rhinitis	87 (34.1)
Gastroesophageal reflux	67 (26.3)
Hypertension	67 (26.3)
Dyslipidemia	42 (16.5)
Anxiety	42 (16.5)
Depression	39 (15.3)
Osteoporosis	37 (14.5)
T2DM	32 (12.5)
Bronchiectasis	32 (12.5)
Obstructive Sleep Apnea Syndrome	26 (10.2)
Aspirin exacerbated respiratory disease	23 (9.0)
Chronic obstructive pulmonary disease	13 (5.1)
CRSsNP	12 (4.7)
Allergic bronchopulmonary aspergillosis	10 (3.9)
Atopic dermatitis	8 (3.1)
Cataracts	4 (1.6)
Eosinophilic granulomatosis with polyangiitis	4 (1.6)
Eosinophilic esophagitis	2 (0.8)
Use of SCS for comorbidities treatment ^g^	26/239 (10.9)

Data are shown as mean (SD) unless otherwise indicated. Due to the nature of this real-world study, sample sizes vary due to missing/unavailable/not valid. * As determined by the investigator. Missing data ^a^ n = 1; ^b^ n = 25; ^c^ n = 62; ^d^ n = 34; ^e^ n = 10; ^f^ n = 12; ^g^ n = 16. BMI = body mass index; CRSsNP = chronic rhinosinusitis without nasal polyps; CRSwNP = chronic rhinosinusitis with nasal polyposis; quartiles 1 and 3 [Q1–Q3]; SCS = systemic corticosteroids; SD = standard deviation; SEA = severe eosinophilic asthma; T2DM = Type 2 Diabetes Mellitus.

**Table 2:** Baseline clinical characteristics of the study population (48 weeks before benralizumab).

Parameters	Total (N = 255)
**Blood eosinophil count** (cells/μL), median [Q1–Q3] ^a^	405 [200–600]
Highest historical eosinophil count ^b^	675 [485–1000]
Highest eosinophil count 48 weeks prior to benralizumab ^c^	430 [205–670]
**Total serum IgE** (IU/L), median [Q1–Q3] K ^d^	157 [52.4–381]
**FeNO** (ppb), median [Q1–Q3] K ^e^	35 [19–65]
**Previous biological treatment** ^f^	
Naïve	154/254 (60.4)
Switch	100/254 (39.4)
**Patients with previous biological treatments** *^,e^	100/254 (39.4)
Omalizumab	72/100 (72.0)
Discontinuation reasons	44/72 (61.1)
*No response*	34 (47.2)
*Loss of response*	25 (34.7)
*Toxicity*	1 (1.4)
*Loss to follow-up*	1 (1.4)
*Other*	9 (12.5)
*N/A*	2 (2.8)
Mepolizumab	44/100 (44.0)
Discontinuation reasons	44/44 (100.0)
*No response*	21 (47.7)
*Loss of response*	14 (31.8)
*Toxicity*	3 (6.8)
*Patient’s decision*	1 (2.3)
*Other*	3 (6.8)
*N/A*	2 (4.5)
Reslizumab	2/100 (2.0)
Discontinuation reasons	2/2 (100.0)
*No response*	1 (50.0)
*Toxicity*	1 (50.0)
**Other asthma treatments** ^f^	
LTRA ^g^	168/254 (66.1)
Corticosteroids	
ICS + LABA ^g^	71/253 (28.1)
ICS + LABA + LAMA ^g^	178/253 (70.4)
SCS ^h^	179/251 (71.3)
median daily SCS use †, median [Q1–Q3] ^c^	1.6 [0.7–3.8]
Cortico-dependent patients	38/255 (14.9)
median daily SCS use †, median [Q1–Q3] ^c^	6.9 [3.5–17.6]
**Severe exacerbations**	
Patients with severe exacerbations	150/255 (58.8)
Severe exacerbations annual rate (per patient-year)	1.55
Use of SCS for treatment	145/255 (56.9)
ER visit	77/255 (30.2)
Hospital admission	26/255 (10.2)
**Pulmonary function tests performed** ^g^	252/253 (99.6)
**Lung function**, mL, mean (SD)	
FEV_1_^i^	1951.4 (804.1)
FEV_1_ predicted ^j^	70.6 (20.7)
Patients with FEV_1_ ≥ 80% ^j^	78 (33.6)
**Asthma control** ^k^	
ACT score, mean (SD) ^l^	13.8 (5.6)
ACT score ≥ 20 ^l^	35/183 (19.1)

Data are shown n/N (%) unless otherwise indicated. Missing data ^a^ n = 12; ^b^ n = 3; ^c^ n = 31; ^d^ n = 67; ^e^ n = 85; ^f^ n = 1; ^g^ n = 2; ^h^ n = 4; ^i^ n = 32; ^j^ n = 20; ^k^ n = 18; ^l^ n = 5. * Patients could have used one or multiple biological therapies before benralizumab initiation. † SCS median daily doses were calculated as prednisone equivalents. ACT = Asthma Control Test; FeNO = fractional exhaled nitric oxide; FEV_1_ = forced expiratory volume in the first second; ICS = inhaled corticosteroids; IgE = Immunoglobulin E; LABA = long-acting beta-agonists; LAMA = long-acting muscarinic antagonists; LTRA = leukotriene receptor antagonists; N/A = not available; quartiles 1 and 3 [Q1–Q3]; ppb = parts per billion; SCS = systemic corticosteroids; SD = standard deviation.

## Data Availability

The raw data supporting the conclusions of this article will be made available by the authors on request, in accordance with AstraZeneca’s data sharing policy, described at https://astrazenecagrouptrials.pharmacm.com/ST/Submission//Disclosure (accessed 19 March 2026).

## Supplementary Materials

The following supporting information can be downloaded at: https://www.mdpi.com/article/10.3390/jcm15124564/s1, Figure S1: Evolution of the benralizumab administration setting during long-term follow up; Figure S2: Global annual rates of ER visits and hospital admissions (patient-years) (A), and of visits regarding asthma specialist and general practitioner (patient-years) (B); Table S1: Reasons for temporary and permanent discontinuation.
