# Supplementary material for: Long-Term Adherence to Benralizumab and Sustained Clinical Benefits in Patients with Severe Eosinophilic Asthma: Insights from GALERNA, a Retrospective Real-World Study in Spain
Source: J Clin Med. 2026 Jun 12;15(12):4564. doi: 10.3390/jcm15124564 (PMC13301468; doi:10.3390/jcm15124564)
Supplement: Supplementary file 1 [file jcm-15-04564-s001.zip › jcm-4350170-supplementary.pdf]

Supplementary Figure S1. Evolution of the benralizumab administration setting during long-term follow up. Percentage of patients receiving benralizumab in a hospital setting or through self-administration at each scheduled visit from treatment initiation (week 0) up to week 144. Percentages are calculated among patients with available data at each time point.

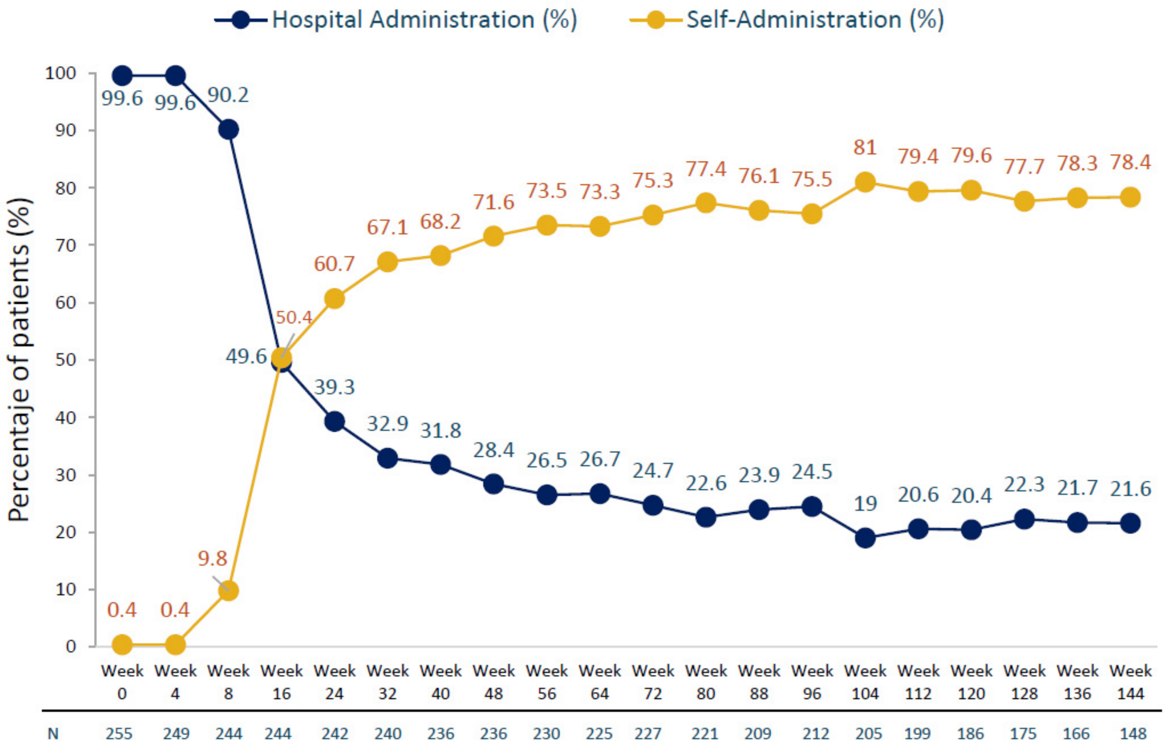

Supplementary Figure S2. Global annual rates of ER visits and hospital admissions (patient-years) (A),and of visits regarding asthma specialist and general practitioner (patient-years) (B). FUP: follow-up period.

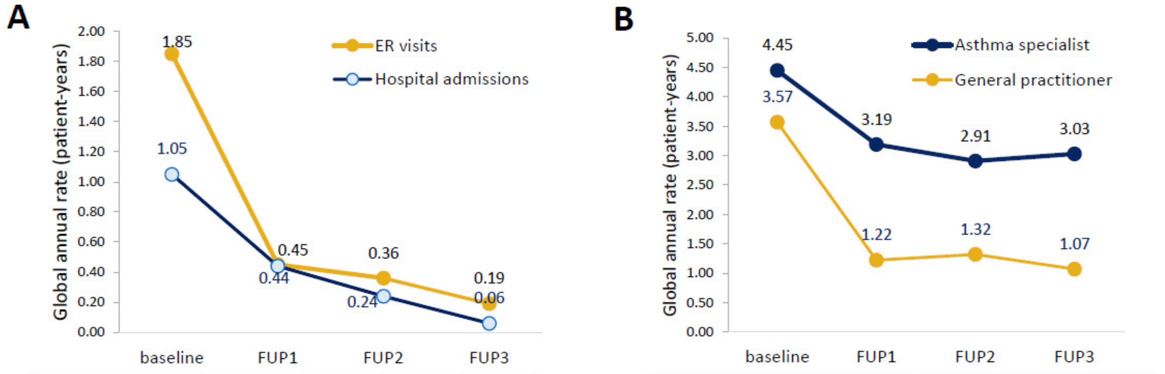

N	baseline	FUP1	FUP2	FUP3
Hospital admissions	38	33	29	26
ER visits	81	81	66	62

N	baseline	FUP1	FUP2	FUP3
Asthma specialist	228	229	208	183
General practitioner	70	70	63	58

Supplementary Table S1: Reasons for temporary and permanent discontinuation.

Main reasons for discontinuation	
[a] **For temporary discontinuations,** n/N (%)	[illegible]54 (5.9)
Adverse event	[illegible].7)
Pregnancy and/or lactation	[illegible]3)
Patient decision	[illegible].3)
Other	[illegible]1.7)
[b] **For permanent discontinuations,** n/N (%)	[illegible]52 (20.2)
Lack of response	[illegible]4.0)
Loss of response	[illegible]2.0)
Adverse event	[illegible]0)
Loss of follow-up	[illegible]0)
Patient decision	[illegible]0)
Death	[illegible]0)
Other	[illegible]2.0)

Missing data: [a] n = 3; [b] n = 1.
